# Acute necrotizing duodenitis in diabetic ketoacidosis

**DOI:** 10.1007/s12024-024-00800-z

**Published:** 2024-10-05

**Authors:** Zeena Gadsby, Melissa Thompson, Rexson Tse

**Affiliations:** 1 School of Medicine and Dentistry, Griffith University, Southport, QLD Australia; 2 Forensic and Scientific Services, Health Support Queensland, Southport, QLD 4215 Australia

**Keywords:** Postmortem, Autopsy, Diabetes, Diabetic ketoacidosis, Acute necrotising duodenitis

## Abstract

Acute necrotizing esophagitis (ANE), acute necrotising duodenitis (AND), and Wishnesky’s lesions (WLs) are three peculiar upper gastrointestinal pathologies that can be seen in death from diabetic ketoacidosis (DKA). Amongst these three, AND has only been recently described. Morphologically, ANE and AND present as generalized black discoloration of the intestinal tract, and florid necrosis and inflammation. Whereas WLs are discrete black lesions in the stomach with necrosis and muted inflammation. We report a case of isolated AND with an unusual morphology not previously reported. A man in his 60s was found dead at home who died from pneumonia complicated by DKA. The gastrointestinal tract showed isolated patchy and discrete AND in which macroscopically resembled WLs, but microscopy resembled ANE with florid necrosis and acute inflammation. This case, together with the literature, documented AND can be macroscopically diffuse or discrete resembling ANE or WLs respectively but microscopically resemble ANE. Furthermore, the potential of these lesions being found in isolation in DKA raises the possibility of both general and local mechanisms playing a role on their morphology and presentation.

## Case report

A man in his 60s was found lying next to his bed deceased at his residence. According to the information provided by the police report of death to the coroner, he suffered from type 2 diabetes and was non-compliant with his medication.

**Fig. 1:**
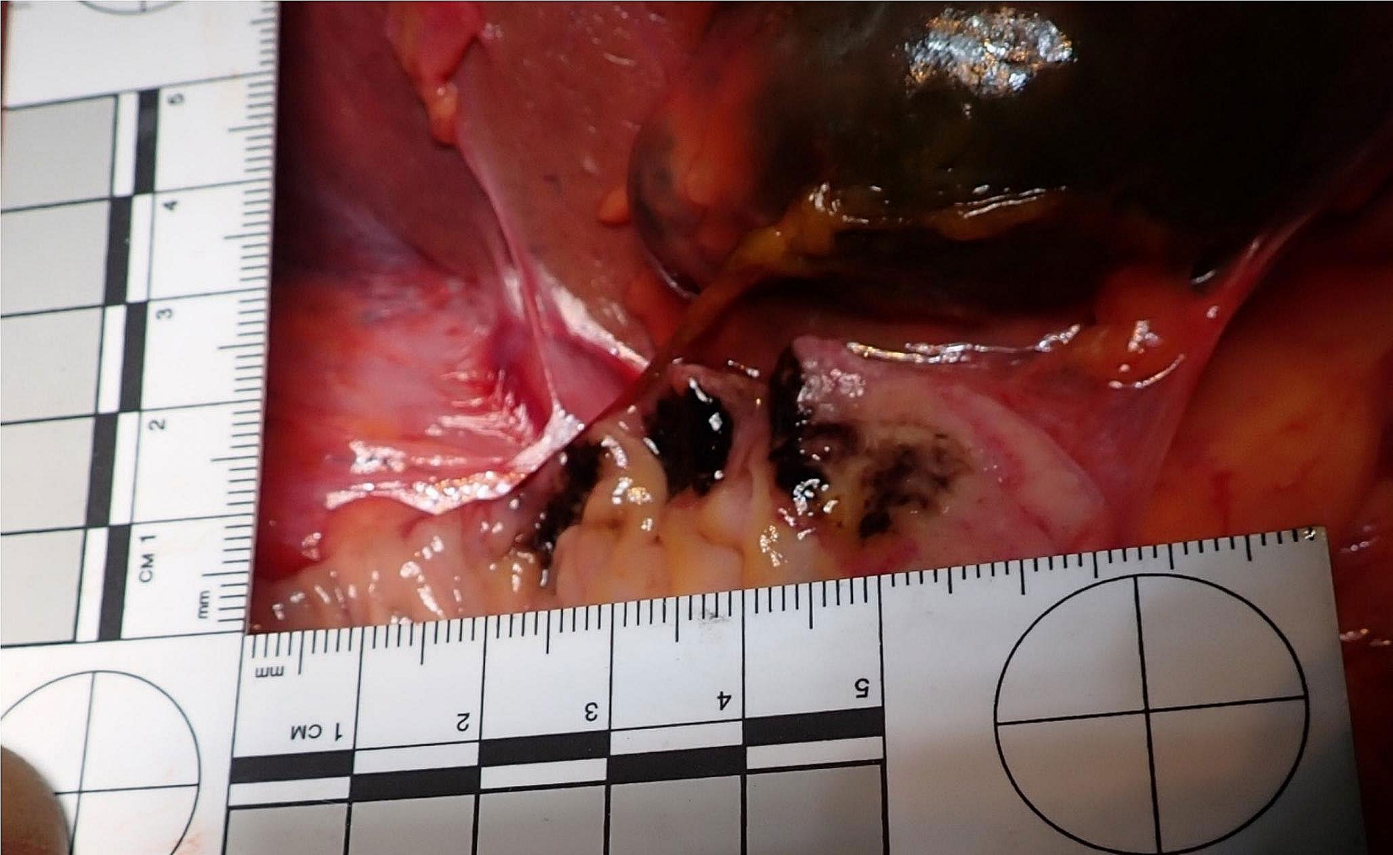
Macroscopic image of the first part of the dissected duodenum, showing clear demarcated black patches proximal to the Ampulla of Vater

**Fig. 2:**
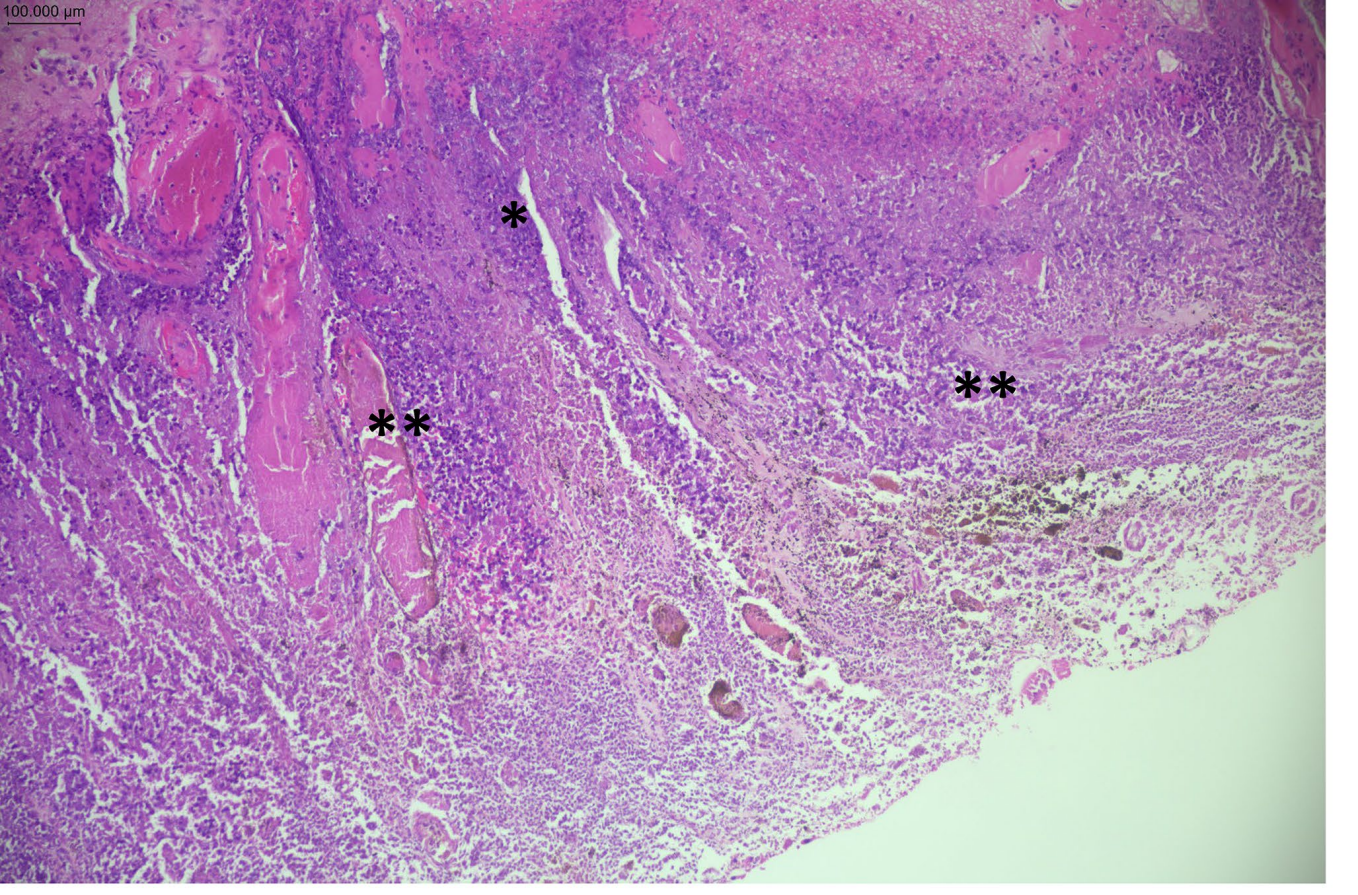
Histological image of biopsy taken from pigmented area in duodenum, showing florid neutrophil infiltration and necrosis (*) and black pigmentation deposition on the mucosa and vascular lining (**) in keeping with acute necrotising duodenitis

Postmortem examination was performed four days after the death. A routine unenhanced postmortem computed tomography scan showed increased opacity in the lungs, liver and a distended bladder. External examination showed a slightly overweight male (weight: 78 kg, height 173 cm) with no injuries on the body. On internal examination, the lungs showed bilateral consolidation in which histopathology confirmed acute bronchopneumonia. The upper gastrointestinal track showed areas of black discolouration in the first part of the duodenum proximal to the Ampula of Vater characterised by three well discrete patches that could not be manually removed or washed off (Fig. 1). Histologically, these areas of the duodenum showed florid neutrophil infiltration, necrosis and black pigmentation deposition on the mucosa and vascular lining (Fig. 2) in keeping with acute necrotising duodenitis (AND).

The stomach and oesophagus were unremarkable with no evidence of inflammation. The pancreas showed fibrosis in keeping with chronic pancreatitis, and the liver showed mild steatosis. Vitreous humour biochemistry showed elevated glucose and β -hydroxybutyrate at 21.2mmol/L and 2.4mmol/L in keeping with diabetic ketoacidosis (DKA).

The cause of death was pneumonia complicating DKA. This man would have had pneumonia and developed an acute metabolic complication from diabetes, namely DKA. It is probable that with type 2 diabetes mellitus and chronic pancreatitis, this man was unable to produce sufficient insulin and developed DKA in the face of increased physiological demand.

## Discussion

This case documented isolated AND (or ‘black duodenum’), in a death from pneumonia complicated by DKA. The peculiar features in this case are not only the isolated presentation but also its overlapping morphology with other upper gastrointestinal lesions associated with DKA, namely acute necrotizing esophagitis (ANE or ‘black esophagus’) and Wishnesky’s lesions (WLs). This case provides insight into the pathogenesis of these peculiar gastrointestinal findings, and documents the variation in presentation of AND in the context of DKA.

Literature described the different morphological appearances between ANE, WLs and AND in their respective locations. Proposed overlapping mechanisms that cause these lesions include general metabolic derangement and local factors such as blood perfusion, and exposure to gastric content (gastric acid and enzymes) [1–4]. Macroscopically, ANE presents as generalised black discolouration of the oesophagus propagating from the distal oesophagus and extending proximally and has a definite demarcation at the gastro-oesophageal junction [4, 5]. WLs are black lesions ranging from punctate dots to up to 40 mm on the gastric mucosa [3, 6]. AND is recently reported in deaths from DKA in two separate case reports (with photograph) and one from sepsis (without photograph) in the forensic pathology literature [1, 7, 8]. The two case reports related to DKA described/documented AND to have the macroscopic appearance resembling ANE with either generalised black discolouration or a singular dark lesion on the duodenum which have a definite demarcation at the gastro-duodenal junction [1, 6, 8, 9]. Furthermore, the AND in both cases were seen with ANE and WLs. In the presented case, the AND was isolated without ANE and WLs, and macroscopically it was discrete and patchy resembling WLs, which differed from what was previously described.

Histologically all three conditions have dark granular collections and necrotic features. Different from WLs, ANE and AND have pronounced neutrophilic and lymphocytic infiltration of the mucosa [1, 6, 9]. Despite the morphology of the duodenal lesions in this case resembling WLs, the histology resembles that of AND in keeping with previous case reports. A summary of the macroscopic and microscopic morphology of these three lesions is shown in Table 1.

**Table 1:** Summary of morphological and histological features of three gastrointestinal features found in diabetic ketoacidosis: acute necrotising esophagitis, Wishnesky’s lesions, and acute necrotising duodenitis

	Acute necrotising esophagitis (ANE)	Wishnesky’s lesions (WLs)	Acute necrotising duodenitis (AND)
Primary location	Distal esophagus	Gastric mucosa	Proximal duodenum
Shape	Diffuse	Discrete lesions	Diffuse or discrete lesions
Dark granules	↑	↑	↑
Necrosis present	Yes	Yes	Yes
Neutrophilic/Lymphocytic infiltration	↑	↓	↑

## Conclusion and recommendation

From the literature and the presented case, it appears that ANE, WLs, and AND can occur in isolation or together with DKA with variable morphologies. The general metabolic derangement and local factors likely plays a role in the varying morphology and presentation of these pathologies.

We hypothesize the under reporting of AND in literature may be due to lack of appreciation as its appearance can be easily discounted by postmortem bile staining of the mucosa [10]. We recommend close examination of the duodenum in cases of suspected underlying metabolic derangement (in particular DKA) and have a low threshold in discounting discoloration in the duodenum and sample for histology to confirm.
